# The Comparative Study of the Effects of Botulinum Toxin Micro-droplet Injection Combined with Micro-crosslinked Sodium Hyaluronate Gel or Focused Ultrasound Technology in Facial Rejuvenation Treatment

**DOI:** 10.1007/s00266-025-04903-y

**Published:** 2025-06-04

**Authors:** Yanan Jiang, Yerong Jiang, Yu Gao, Lu Yan, Demei Zhao

**Affiliations:** 1 Department of Burns and Plastic Surgery, The Affiliated Hospital of Nanjing University Medical School, Nanjing Drum Tower Hospital, Nanjing, People’s Republic of China; 2 Medical Cosmetic Center, The Affiliated Hospital of Nanjing University Medical School, Nanjing Drum Tower Hospital, Nanjing, People’s Republic of China

**Keywords:** Botulinum toxin micro-droplet injection, Micro-crosslinked sodium hyaluronate gel, Focused ultrasound technology, Facial rejuvenation treatment

## Abstract

**Background:**

The harmonization of micro-droplet botulinum toxin injections with either micro-crosslinked sodium hyaluronate gel or focused ultrasound technology necessitating a comprehensive evaluation of their differential impact.

**Methods:**

A retrospective analysis of clinical data was conducted on 114 patients who underwent facial rejuvenation treatment. The patients were divided into two groups: one receiving botulinum toxin micro-droplet injection combined with micro-crosslinked hyaluronic acid sodium gel (*n*=56) and the other receiving botulinum toxin micro-droplet injection combined with focused ultrasound technology (*n*=58). The study assessed baseline characteristics, procedural aspects, recovery, side effects, facial laxity and rejuvenation (FLR), and quality of life using validated rating scales and criteria.

**Results:**

The study revealed several significant findings. The procedural duration was shorter in the micro-crosslinked sodium hyaluronate gel group, whereas the time to return to normal activities was shorter in the focused ultrasound group. The incidence of edema and erythema was significantly lower in the focused ultrasound group. FLR scale scores showed better skin elasticity and tightness in the focused ultrasound group, while quality of life scores indicated variations in emotion management and return to social function between the two groups.

**Conclusion:**

The study emphasizes the multifaceted impact of botulinum toxin micro-droplet injection combined with micro-crosslinked sodium hyaluronate gel and focused ultrasound technology on various parameters in facial rejuvenation treatment. These findings provide valuable insights into the differential effects of these treatment modalities, highlighting the need for personalized approaches and patient-centered care in cosmetic dermatology and plastic surgery.

**Level of Evidence II:**

This journal requires that authors assign a level of evidence to each article. For a full description of these Evidence-Based Medicine ratings, please refer to the Table of Contents or the online Instructions to Authors  www.springer.com/00266.

## Introduction

Facial rejuvenation has become increasingly popular in the field of aesthetic medicine and plastic surgery, driven by the growing demand for noninvasive or minimally invasive procedures aimed at enhancing facial aesthetics and ameliorating the visible signs of aging [1, 2]. Among the diverse array of available treatment modalities, botulinum toxin micro-droplet injection combined with micro-crosslinked sodium hyaluronate gel and focused ultrasound technology have emerged as prominent options for addressing facial laxity, wrinkles, and overall skin rejuvenation [3–5]. The use of botulinum toxin for facial rejuvenation has been well established, with its mechanism of action involving the selective inhibition of acetylcholine release at the neuromuscular junction, leading to muscle relaxation and subsequent improvement in the appearance of dynamic wrinkles and facial lines [6–8]. In recent years, micro-droplet injection techniques have gained traction as a means of achieving a more subtle and natural aesthetic outcome, targeting not only dynamic wrinkles but also providing overall skin tightening and rejuvenation effects [9–11]. Concurrently, the utilization of micro-crosslinked sodium hyaluronate gel as a dermal filler has been recognized for its ability to enhance facial volume, improve skin texture, and promote tissue hydration, thereby contributing to a comprehensive approach to facial rejuvenation [12–14].

In parallel, focused ultrasound technology has garnered attention as a noninvasive modality for facial skin tightening and contouring, utilizing targeted ultrasound energy to induce collagen denaturation and neocollagenesis within the deep dermal layers, thereby promoting tissue remodeling and firming [15, 16]. The precision and depth control afforded by focused ultrasound technology offer distinct advantages in addressing facial laxity, particularly in regions prone to skin sagging and loss of elasticity [17–19]. Despite the growing utilization of these treatment modalities, direct comparative studies evaluating their respective effects and outcomes in facial rejuvenation were relatively scarce in the current literature. The harmonization of micro-droplet botulinum toxin injections with either micro-crosslinked sodium hyaluronate gel or focused ultrasound technology represents a point of therapeutic confluence, necessitating a comprehensive evaluation of their differential impact on various aspects of facial aesthetics, patient satisfaction, safety, and quality of life [20, 21]. Thus, the present study aims to address this critical knowledge gap by conducting a comparative assessment of the effects of botulinum toxin micro-droplet injection combined with micro-crosslinked sodium hyaluronate gel and focused ultrasound technology in facial rejuvenation treatment.

## Methods

### Study Design and Population

This study conducted a retrospective analysis of clinical data of patients who underwent facial rejuvenation treatment at our hospital from January 2023 to December 2023, totaling 114 cases. Based on different treatment modalities, the patients were divided into the group receiving micro-droplet injection of botulinum toxin combined with micro-crosslinked hyaluronic acid sodium gel (*n*=56) and the group receiving micro-droplet injection of botulinum toxin combined with focused ultrasound technology (*n*=58). This study was approved by our hospital Institutional Review Board and Ethics Committee. Informed consent was waived for this retrospective study due to the exclusive use of de-identified patient data, which posed no potential harm or impact on patient care.

The inclusion criteria were as follows: Patients who underwent facial rejuvenation treatment at our hospital between January 2023 and December 2023. All the patients aged 18 years or older. Patients who received either micro-droplet injection of botulinum toxin combined with micro-crosslinked sodium hyaluronate gel or micro-droplet injection of botulinum toxin combined with focused ultrasound technology for facial rejuvenation. The following criteria will result in exclusion from the study: Patients with incomplete or missing clinical data. Patients who underwent concurrent facial rejuvenation procedures or treatments other than the specified modalities under investigation during the study period. Patients with a history of significant dermatological or systemic diseases that could impact the outcomes of the facial rejuvenation procedures. Patients with a history of adverse reactions or allergies to the components of the treatment modalities. Patients who were unable to provide informed consent or comply with the follow-up schedule.

### Micro-droplet Injection of Botulinum Toxin

**Fig. 1:**
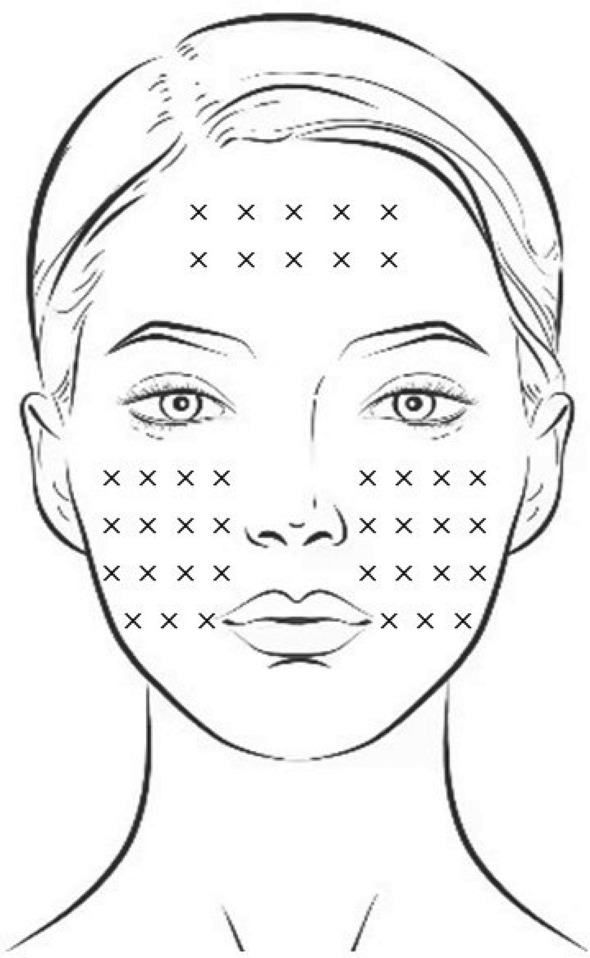
Schematic diagram of micro-droplet injection of botulinum toxin in the face

A vial of type A botulinum toxin 100U (Botox, Allergan Aesthetics an AbbVie company) was taken for injection. Prior to injection, it was diluted with 2.5 ml of 0.9% saline solution, ensuring gentle agitation along the vial wall to avoid excessive air bubbles. 0.5 ml (20U) was drawn from the vial and then diluted with an additional 0.5 ml of 0.9% saline solution to achieve a concentration of 20U/ml botulinum toxin. The treatment area's skin was disinfected with iodine, followed by the administration of 1 ml of the prepared micro-droplet injection solution of type A botulinum toxin (Microbotox). An assistant tightened the skin in the injection area, and a 32G 4 mm needle was used to inject the skin surface. Resistance was felt while administering the drug, and immediate whitish papules were observed on the skin at the injection site, indicating the appropriate injection depth. The distance between points was 0.8–1 cm, with 0.5U injected per point, resulting in the uniform distribution of small papules. Injections were standardized across face in all patients (Fig. 1).

### Treatment with Micro-crosslinked Sodium Hyaluronate Gel

Micro-crosslinked sodium hyaluronate gel (water, 2 ml, Lu Food and Drug Administration Medical Device Production License 20120123, Huaxi Biotechnology Co., LTD.) is 1:1 diluted with 0.9% normal saline. The Derma Shine injection instrument (Model: second generation) is used for treatment. Before treatment, the patient is helped to maintain a supine position and clean the facial skin. Before treatment, apply lidocaine cream (Tongfang Pharmaceutical Group Co., LTD., National Drug Approval number H20063466) to the treatment area, and then wrap with plastic wrap for about 1 hour. Remove the anesthetic, disinfect with iodophor, and then wipe with saline. Then, 95% ethanol was used to sterilize the water needle handle and the negative pressure suction catheter. After the completion, the needle of the water light injection instrument was installed with sterile gloves and connected with the negative pressure suction device. Sodium hyaluronate complex solution for injection, total injection of 4 ml. During treatment, adjust the length of the needle 1-1.5 mm, and then wipe it with normal saline.

### Focused Ultrasound Technology

The MFUS One peninsula ultrasound therapeutic apparatus (MFUS One, Hunan Peninsula Medical Technology Co., Ltd.) was employed, and different treatment heads (M4.5, M3.0, D4.5, D3.0) were sequentially selected according to the operational procedures. The treatment steps were as follows: (1) instruct the patient to cleanse their face and take photos; (2) connect the apparatus and install the treatment head; (3) inspect and confirm the treatment position, then use a white marking pen to clearly outline each area; (4) uniformly apply coupling agent to the operating area, flatten the skin to ensure close contact between the treatment head and the skin. The M3.0 and M4.5 heads were used in position-stamping mode at level IV energy, controlled with the handle to emit energy, with the treatment head moved approximately 1 mm upward after each emission and then proceeding with the treatment. The D4.5 and D3.0 heads were operated in sliding mode at level V energy. After treatment, strict sun protection and moisturizing were recommended. A single treatment of focused ultrasound is about 60 minutes.

### Wrinkle Severity

The Facial Contour Tightness Assessment Scale (Cronbach’s *α*=0.769) was used to evaluate the patients'facial wrinkle severity, including facial wrinkle condition, mandibular shape, and nasolabial groove morphology, with each item rated on a five-point scale (0–4 points) based on aesthetic appearance. The total score, ranging from 0 to 12 points, indicates the higher the score, the better the facial aesthetic appearance.

### Satisfaction

Assessment was conducted using a custom satisfaction scale from our hospital, with a total score of 10 points. A score of ≥9 points indicated “very satisfied,” while satisfaction was categorized as “satisfied” for scores between 7 and 9 points, “fairly satisfied” for scores between 6 and 7 points, and"unsatisfied"for scores less than 6 points. A higher satisfaction score indicated a higher level of patient satisfaction.

### Quality of Life Assessment

The SF-36 Health Survey (Short Form 36) was a commonly used tool for assessing health-related quality of life applicable to diverse populations. Its purpose was to understand an individual’s physical functioning, mental health, social functioning, and overall health status. This tool comprises eight health-related quality of life questionnaires, encompassing physical functioning, role limitations due to physical health, role limitations due to emotional problems, mental health, social functioning, energy/fatigue, and general health perception. Each question was rated on a five-point scale, ranging from “excellent,” “very good,” “good,” “fair,” to “poor.” Scores for each question range from 0 to 100, where higher scores indicate a lesser impact of the given issue.

### FLR Scale Scores

This was a scoring system utilized to assess the degree of facial skin laxity and reshaping. The system primarily involves the physician's observation of the patient's facial characteristics, combined with objective standards, to assign scores based on the degree of laxity, wrinkles, and reshaping needs in five different facial areas. This scoring system aids in providing a systematic evaluation, facilitating a better understanding of the facial skin condition for both physicians and patients. A higher score indicates a greater degree of skin reshaping. The scores on the FLR Scale are categorized as follows: 0 points for absent; 1 point for mild; 2 points for noticeable; 3 points for marked; 4 to 6 points for moderate; and 7 to 9 points for severe.

### Data Collection

Baseline characteristics, including age, gender, Fitzpatrick skin type, duration of wrinkles, and baseline wrinkle severity, were recorded for all participants. Additionally, procedural aspects such as procedure duration, time to return to normal activities, downtime >3 days, and need for touch-up treatments were documented.

### Statistical Analysis

The data were analyzed using SPSS 29.0 statistical software (SPSS Inc., Chicago, IL, USA). For categorical data, [*n*(%)] was used for representation. The Chi-square test was applied with the basic formula when the sample size was ≥ 40 and the theoretical frequency *T* was ≥ 5, with the test statistic represented by χ^2^. When the sample size was ≥ 40 but the theoretical frequency 1 ≤ *T* < 5, the Chi-square test was adjusted using the correction formula. In cases where the sample size was < 40 or the theoretical frequency *T* < 1, statistical analysis was conducted using Fisher's exact probability method. For normally distributed continuous data, the format as mean ± standard deviation (SD) was employed. Non-normally distributed data were analyzed using Wilcoxon rank-sum test. *P* < 0.05 were considered as statistical significance.

## Results

### Baseline Characteristics

The baseline characteristics of the participants in this comparative study of the effects of botulinum toxin micro-droplet injection combined with micro-crosslinked sodium hyaluronate gel or focused ultrasound technology in facial rejuvenation treatment were well balanced between the two groups (Table 1). The mean age of participants in the botox + hyaluronate gel group was 40.25 ± 5.33 years, while it was 41.15 ± 4.53 years in the botox + focused ultrasound group (*P >* 0.05). Additionally, there were no significant differences between the two groups in terms of Fitzpatrick skin type, duration of wrinkles, and baseline wrinkle severity (*P >*0.05), as indicated by the nonsignificant t test results and p values for these parameters.

**Table 1:** Baseline characteristics of participants

Parameters	Botox + hyaluronate gel group (*n*=56)	Botox + focused ultrasound group (*n*=58)	t/χ^2^	*P*
Age (years)	40.25 ± 5.33	41.15 ± 4.53	0.971	0.334
Fitzpatrick skin type	2.51 ± 0.54	2.63 ± 0.65	1.088	0.279
Duration of wrinkles (years)	4.82 ± 1.26	4.58 ± 1.34	1.018	0.311
Baseline wrinkle severity	7.23 ± 0.87	7.36 ± 0.85	0.776	0.44

### Duration of Procedure and Recovery

The comparison of procedural aspects and recovery parameters between the botox + hyaluronate gel and botox + focused ultrasound groups revealed several significant findings (Table 2). The procedure duration was shorter in the botox + hyaluronate gel group compared to the botox + focused ultrasound group (*P* < 0.001). Additionally, edema resolution time was significantly longer in the botox + hyaluronate gel group compared to the botox + focused ultrasound group (*P* < 0.001). The percentage of patients and the need for touch-up treatments were also significantly different between the two groups, with lower percentages in the botox + focused ultrasound group (*P =* 0.004). However, there was no significant difference in overall recovery satisfaction between the two groups (*P =* 0.862).

**Table 2:** Duration of procedure and recovery

Parameters	Botox + hyaluronate gel group (*n*=56)	Botox + focused ultrasound group (*n*=58)	t	*P*
Procedure duration (minutes)	35.55 ± 5.46	81.27 ± 5.71	43.697	< 0.001
Edema resolution time (d)	6.26 ± 1.53	5.08 ± 1.75	3.82	< 0.001
Need for touch-up treatments (%)	15.42 ± 5.32	12.12 ± 6.53	2.965	0.004
Overall recovery satisfaction	9.21 ± 1.06	9.17 ± 1.14	0.174	0.862

### Side Effects and Complications

**Fig. 2:**
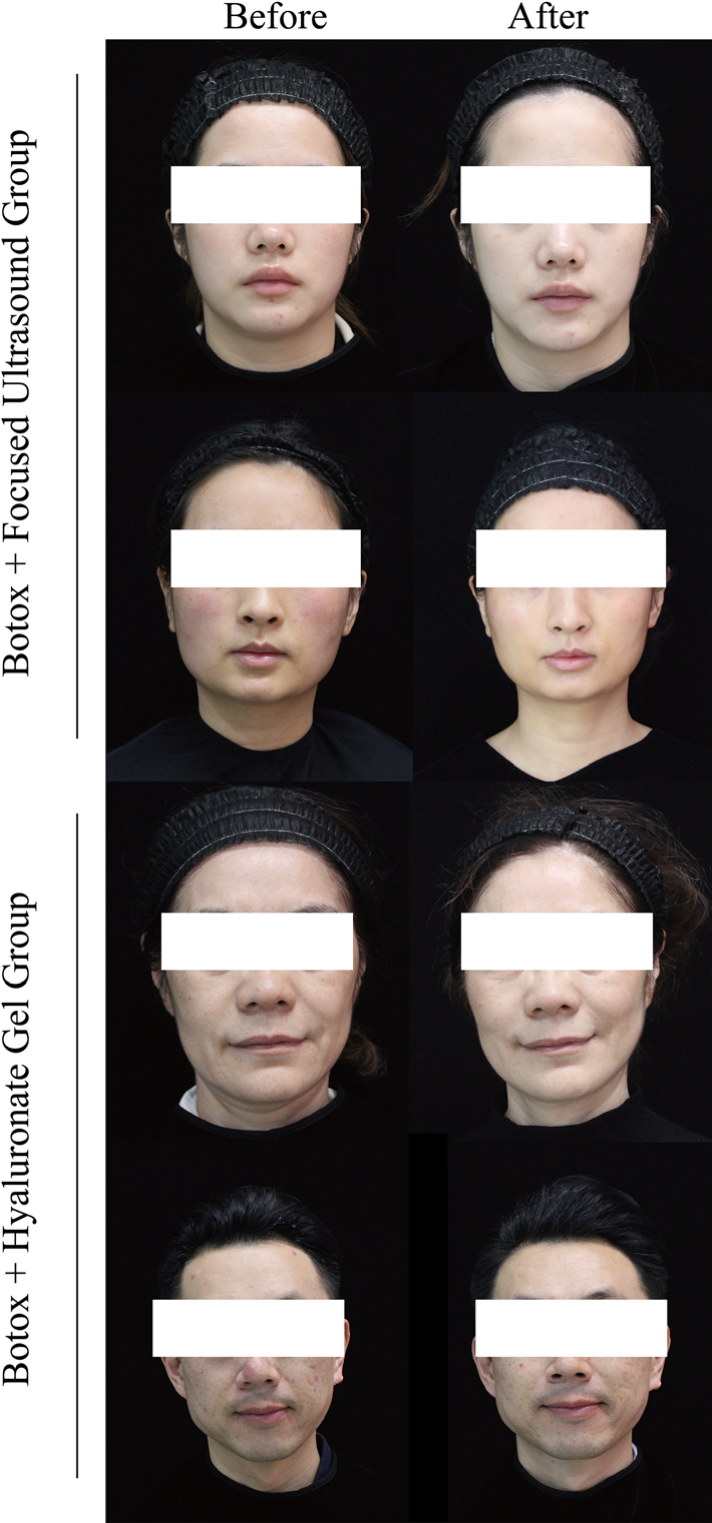
Pre- and post-treatment images of representative patients, including botox + focused ultrasound group and botox + hyaluronate gel group

In the comparative study of side effects and complications between the botox micro-droplet injection combined with micro-crosslinked sodium hyaluronate gel group and the botox micro-droplet injection combined with focused ultrasound technology group, the incidence of edema was significantly higher in the botox + hyaluronate gel group as was the incidence of erythema. There were no significant differences between the two groups in terms of numbness or bruising (Table 3). Pre- and post-treatment images of representative patients are presented in Fig. 2.

**Table 3:** Side effects and complications

Parameter	Botox + hyaluronate gel group (*n*=56)	Botox + focused ultrasound group (*n*=58)	χ^2^	*P*
Edema (n, %)	17 (29.31%)	5 (8.93%)	6.347	0.012
Erythema (n, %)	16 (27.59%)	4 (7.14%)	6.879	0.009
Pain (n, %)	3 (5.36%)	5 (8.62%)	0.099	0.753
Bruising (n, %)	4 (7.14%)	6 (10.34%)	0.075	0.785

### FLR Scale Scores

In evaluating the FLR Scale scores at 3 months, significant differences were observed between the botox + hyaluronate gel group and the botox + focused ultrasound group (Tables 4 and 5). The botox + hyaluronate gel group exhibited lower scores for skin elasticity compared to the botox + focused ultrasound group. Additionally, the botox + hyaluronate gel group had lower scores for skin tightness compared to the botox + focused ultrasound group. While the differences in facial wrinkles scores were statistically significant, the clinical significance may require further investigation, with the botox + focused ultrasound group demonstrating slightly higher scores than the botox + hyaluronate gel group.

**Table 4:** FLR scale scores at 12 months

Parameter	Botox + hyaluronate gel group (n=56)	Botox + focused ultrasound group (n=58)	t	*P*
Skin elasticity	2.56 ± 0.42	3.17 ± 0.39	7.964	< 0.001
Facial wrinkles	3.89 ± 0.68	4.25 ± 0.71	2.762	0.007
Skin tightness	3.32 ± 0.49	2.78 ± 0.52	5.690	< 0.001

**Table 5:** Clinical improvement scores at 12 months

Parameter	Botox + hyaluronate gel	Botox + focused ultrasound	*P*
Wrinkle reduction (%)	38.2 ± 6.7	42.5 ± 5.9	0.012
Skin elasticity improvement	2.8 ± 0.5	3.5 ± 0.6	<0.001
Patient satisfaction (VAS)	8.7 ± 1.1	9.3 ± 0.9	0.003

### Quality of Life

The assessment of quality of life scores demonstrated significant variations between the two study groups (Table 6). Specifically, in the domain of emotion management, the botox + focused ultrasound group exhibited notably higher scores compared to the botox + hyaluronate gel group. Furthermore, the return to social function scores was significantly higher in the botox + hyaluronate gel group compared to the botox + focused ultrasound group. Conversely, no significant differences were observed in the somatization, role play, and cognitive function scores between the two groups. These findings highlight the potential influence of the treatment modalities on specific aspects of patients’ quality of life.

**Table 6:** Quality of life scores

Parameter	Botox + hyaluronate gel group (n=56)	Botox + focused ultrasound group (n=58)	t	*P*
Somatization	82.03 ± 4.02	81.72 ± 3.98	0.407	0.685
Emotion management	78.14 ± 4.78	83.14 ± 5.26	5.313	< 0.001
Role play	85.25 ± 6.23	84.41 ± 5.26	0.778	0.439
Cognitive function	83.21 ± 5.11	84.73 ± 5.02	1.604	0.111
Return to social function	81.73 ± 5.78	79.07 ± 6.23	2.357	0.02

## Discussion

Facial rejuvenation treatments were a rapidly evolving area of cosmetic dermatology and plastic surgery, with diverse modalities aiming to address the signs of aging and enhance facial aesthetics [22, 23]. Among the various treatment options, botulinum toxin micro-droplet injection combined with micro-crosslinked sodium hyaluronate gel and focused ultrasound technology have gained momentum due to their efficacy in promoting skin tightening and rejuvenation [24–26]. In this comparative study, we sought to evaluate the effects of these two modalities in facial rejuvenation treatment, encompassing various parameters including baseline characteristics, procedural aspects, recovery, side effects, FLR, and quality of life.

The differential effects of botulinum toxin micro-droplet injection combined with micro-crosslinked sodium hyaluronate gel and focused ultrasound technology in facial rejuvenation treatment can be attributed to several underlying factors. Botulinum toxin micro-droplet injection selectively inhibits acetylcholine release at the neuromuscular junction, resulting in muscle relaxation and improvement in the appearance of dynamic wrinkles and facial lines. Conversely, micro-crosslinked sodium hyaluronate gel acts as a dermal filler, enhancing facial volume, improving skin texture, and promoting tissue hydration. Meanwhile, focused ultrasound technology induces collagen denaturation and neocollagenesis in the deep dermal layers, leading to tissue remodeling and firming. These distinct mechanisms target specific aspects of facial aging and rejuvenation, culminating in varied outcomes. The precision of focused ultrasound technology in targeting specific dermal layers prone to skin laxity and loss of elasticity contributes to its effects on skin firmness and tightness. In contrast, micro-crosslinked sodium hyaluronate gel primarily impacts superficial and mid-dermal layers, influencing skin hydration and volume restoration. Furthermore, focused ultrasound technology stimulates neocollagenesis and tissue remodeling within the deep dermal layers, promoting structural support and firmness, while micro-crosslinked sodium hyaluronate gel contributes to volumization and hydration, influencing facial volume and overall skin texture. These differential impacts on tissue stimulation and remodeling contribute to the distinct effects on skin elasticity and tightness observed between the two modalities.

Overall, the comparative analysis of botulinum toxin micro-droplet injection combined with micro-crosslinked sodium hyaluronate gel and focused ultrasound technology in facial rejuvenation treatment provides valuable insights into the multifaceted effects of these modalities [27, 28]. The assessment of side effects and complications revealed notable differences between the two treatment groups. Specifically, the incidence of edema and erythema was significantly lower in the focused ultrasound group compared to the botulinum toxin micro-droplet injection combined with micro-crosslinked sodium hyaluronate gel group, indicating a potentially better tolerability profile for the former modality. These findings underscore the importance of considering not only the efficacy but also the safety and tolerability of different facial rejuvenation treatments, especially in the context of cosmetic procedures where patient satisfaction and safety were paramount. Evaluation of the FLR Scale scores at 12 months revealed distinct differences between the two treatment groups. The botox microdrop injection combined with focused ultrasound group scored higher on skin elasticity and skin firmness, indicating potentially superior results in skin firmness and youth. However, it was noteworthy to interpret the slightly higher facial wrinkles scores in the focused ultrasound group cautiously, as the clinical significance of this difference warrants further investigation. These findings highlight the multifaceted impact of different treatment modalities on various aspects of facial aesthetics and skin quality, underscoring the need for tailored approaches based on specific patient goals and characteristics.

Significant variations were observed in quality of life scores between the two treatment groups, particularly in the domains of emotion management and return to social function. The group receiving botulinum toxin micro-droplet injection combined with micro-crosslinked sodium hyaluronate gel exhibited higher scores for return to social function, suggesting a potential influence of this modality on the social and psychological well-being of patients undergoing facial rejuvenation. Conversely, the focused ultrasound group demonstrated notably higher scores in emotion management, indicating possible differential effects on emotional well-being and coping strategies following the respective treatments. These findings underscore the comprehensive impact of facial rejuvenation treatments on patients’ quality of life beyond purely aesthetic outcomes, emphasizing the need for holistic assessment and patient-centered care in cosmetic dermatology. These findings contribute to the growing body of evidence guiding the selection of optimal treatment approaches in facial rejuvenation, despite the limitations of selection bias. This study has several limitations. First, its retrospective design may introduce selection bias. Second, the follow-up period was limited to 12 months; longer-term outcomes remain to be explored. Third, the absence of patient-reported aesthetic satisfaction scales beyond institutional tools may affect generalizability. Future prospective studies with larger cohorts and extended follow-ups are warranted. Further prospective studies with larger sample sizes and longer follow-up periods are required to validate and extend these preliminary findings, with the aim of fostering evidence-based decision-making and personalized treatment strategies in cosmetic dermatology and plastic surgery.

## Conclusion

In conclusion, this study underscores the multifaceted impact of botulinum toxin micro-droplet injection combined with micro-crosslinked sodium hyaluronate gel and focused ultrasound technology on various parameters including procedural efficiency, recovery, safety, skin rejuvenation, and quality of life. These findings provide valuable insights into the differential effects of these treatment modalities, emphasizing the need for tailored approaches and patient-centered care in facial rejuvenation treatment.

## Data Availability

All data generated or analyzed in this study are included in the present manuscript.
